# The efficacy and safety of minocycline, metronidazole, ivermectin, and azelaic acid in moderate-to-severe papulopustular rosacea: A systematic review and network meta-analysis

**DOI:** 10.1016/j.jdin.2023.12.010

**Published:** 2024-01-13

**Authors:** Esraa A. Shaheen, Yara E. Aljefri, Abdullah A. Ghaddaf, Khalid M. Alshareef, Abeer K. Alhindi, Narin F. Alanazi, Anwar R. Alrashidi, Abdulhadi Jfri

**Affiliations:** a College of Medicine, King Saud bin Abdulaziz University for Health Sciences, Jeddah, Saudi Arabia; b King Abdullah International Medical Research Center, Jeddah, Saudi Arabia; c College of Medicine, Princess Nourah Bint Abdul Rahman University, Riyadh, Saudi Arabia; d Department of Dermatology, King Fahad Medical City, Riyadh, Saudi Arabia

**Keywords:** azelaic acid, ivermectin, metronidazole, minocycline, papulopustular rosacea

## Abstract

**Background:**

There are multiple topical agents for papulopustular rosacea (PPR), but the most effective for the management of moderate-to-severe PPR remains unknown.

**Objective:**

To compare the efficacy and safety of topical agents for moderate-to-severe PPR.

**Methods:**

Medline, Embase, and CENTRAL databases were searched. The efficacy of topical agents was explored through frequentist network meta-analysis using random-effects model. Treatments were ranked using net rank function, yielding P scores.

**Results:**

Nineteen randomized control trials (RCTs) that enrolled and 8208 participants were deemed eligible. Azelaic acid 20% yielded the highest effect size (OR = 8.54, 95% CI: 2.48-29.45) and highest P-score (P score = 0.97) with respect to improvement in investigator global assessment (IGA) score. Azelaic acid 15%, Metronidazole 0.75%, and Ivermectin 1% yielded comparable effect sizes. Azelaic acid 15% yielded statistically significant odds ratio (OR = 1.95, 95% CI: 1.30-2.93, P score = 0.14) for adverse events. Adverse event risk for other topical agents was not significant.

**Limitation:**

The sample size was limited for some of the topical agents. Also, many clinically important outcomes were overlooked by most of the included RCTs.

**Conclusion:**

Azelaic acid 20% was the most effective in improving IGA score for moderate-to-severe PPR and azelaic acid 15% as having the highest adverse event profile.

**Capsule Summary**

• Our network meta-analysis ranked the most effective topical agents for the management of moderate-to-severe papulopustular rosacea.
• Dermatologists may consider Azelaic acid 20% as the most effective topical agent for moderate-to-severe papulopustular rosacea.

## Introduction

Rosacea is a chronic skin condition that manifests as recurrent inflammatory lesions.^1^ A systematic review with meta-analysis of 41 patient cohorts covering a total number of 26 million persons found that the prevalence of rosacea ranges between 0.09% and 22.41% with an average of 5.46%.^2^ Although the etiology of rosacea remains to be fully elucidated, certain triggers including neutrophils, keratinocytes, mast cells, or T-helper cells are believed to activate cells and release mediators of inflammation, leading to inflammatory papules and/or pustules, telangiectasias, or hyperplasia of the connective tissue most often pronounced on the central face.^3^^,^^4^ According to The National Rosacea Society, rosacea is classified into 4 subtypes (erythematotelangiectatic, papulopustular, phymatous, and ocular) and 1 variant (lupoid or granulomatous rosacea).^5^

Rosacea is often associated with a substantial impact on health-related quality of life (HRQOL). The psychological stigma of the physical appearance among patients with rosacea has been associated with higher incidence of embarrassment and low self-esteem.^6^ Therefore, treatment is crucial to stabilize the disease and to achieve patient satisfaction. There are a variety of topical agents used for the management of papulopustular rosacea (PPR), but the United States Food and Drug Administration (FDA)-approved and most commonly prescribed topicals are metronidazole (0.75% gel, cream, lotion, and 1% cream gel), azelaic acid (15% gel or 20% cream), and ivermectin (1% cream).^7^, ^8^, ^9^ These topical agents are often used for mild to moderate PPR. Recently, topical minocycline 1.5% foam has also received FDA approval for the management of moderate to severe PPR.^9^ Many systematic reviews have compared topical agents for the management of PPR, but to our knowledge, none have conducted a network meta-analysis to draw insights about the most effective topical agent for the management of moderate to severe PPR.^10^^,^^11^ The aim of this systematic review and network meta-analysis is to address this gap in research and to compare the efficacy and safety of the commonly prescribed topicals including metronidazole, azelaic acid, ivermectin, and minocycline, for the treatment of moderate to severe PPR.

## Methods

We followed the Preferred Reporting Items for Systematic Reviews and Meta-Analyses (PRISMA) and the extension statement for network meta-analysis. This systematic review and network meta-analysis protocol were registered at PROSPERO (CRD42022355230).^12^

### Study selection

The studies of interest were randomized controlled trials (RCTs) published in English only.

The patient population of interest was adults (≥18 years of age) with moderate-to-severe facial PPR defined as Investigators’ Global Assessment (IGA) score of 3 (moderate) or 4 (severe).^13^ The interventions included were topical minocycline foam 1% vs 1.5% vs 3%; metronidazole gel or cream 1% vs 0.75%; azelaic acid 15% vs 20%; and ivermectin 1%, while the control was topical vehicle (placebo) or another topical agent (ie, minocycline, metronidazole, azelaic acid, or ivermectin) that is considered by the included studies as a control arm. Studies including participants with concurrent use of topical or oral medications for rosacea, patients with mild forms of rosacea, or who have other types of rosacea (erythematotelangectatic, phymatous, or ocular rosacea) were excluded. Moreover, phase I RCTs and non-RCT study designs and conference abstracts/oral presentations were excluded. The outcomes of interest for the proportion of individuals achieving “success” were IGA score (ie, IGA score ≤1), inflammatory lesion count, Rosacea Quality of Life Index (RosaQoL) score, facial local tolerability, patient satisfaction, and adverse events.

### Data sources

We searched Medline, Embase, and Cochrane Central Register of Controlled Trials (CENTRAL) until 11 July 2023. The references and citations of the included trials were also searched for relevant studies. The complete search strategy is shown in the Supplementary Material, available via Mendeley at https://doi.org/10.17632/d6x3ysbytf.3

Two independent reviewers screened titles, and abstracts, read the full texts, and extracted the data from the included studies. A third senior reviewer subsequently validated the data extraction and resolved any discrepancies.

### Data extraction and risk of bias

The data extraction was performed using Excel and the following data was extracted from each eligible trial: name of the first author and year of publication; study arms and concentrations; number of participants in each arm; age; gender; baseline IGA score; inflammatory lesion counts; skin type (ie, Fitzpatrick I-VI); duration of the topical treatment; duration of follow up; and the desired outcomes reported by each trial. We used the modified Cochrane Collaboration assessment tool to assess the risk of bias of the eligible studies and classified them into the following categories: high risk of bias, some concerns, or low risk of bias.^14^

### Network meta-analysis

For each study, binary data (events and sample size) were extracted for the intervention and control group. These binary data were used to calculate effect sizes presented as odds ratio (OR). Efficacy of topical agents including minocycline, ivermectin, metronidazole, and azelaic acid for treating PPR was explored using frequentist network meta-analysis, using the Netmeta statistical package in R software. Prior to running network meta-analysis, the assumptions of transitivity were explored among the included trials. Within-design and between-design inconsistencies were quantified using I2 and Cochran’s Q statistic and full design-by-treatment interaction random-effects model. In cases of statistically significant tests of heterogeneity, random-effects models were used. Further consistency checks included evaluating differences between effect estimates based on direct and indirect evidence. Using dmetar package, a direct evidence plot was used to visualise the proportion of direct and indirect evidence for each comparison. All treatments were ranked using the netrank function, yielding P scores which is the proportion out of 1 that represents the probability of each treatment of being the best. This means the higher the proportion the better the ranking. The ranking of treatments was further corroborated by visualising a forest plot using placebo as a reference group. We adopted 95% as a significance level. Egger’s test for funnel plot used for asymmetry due to small-study effects in our networks.

## Results

### Study selection and network structure

**Fig 1:**
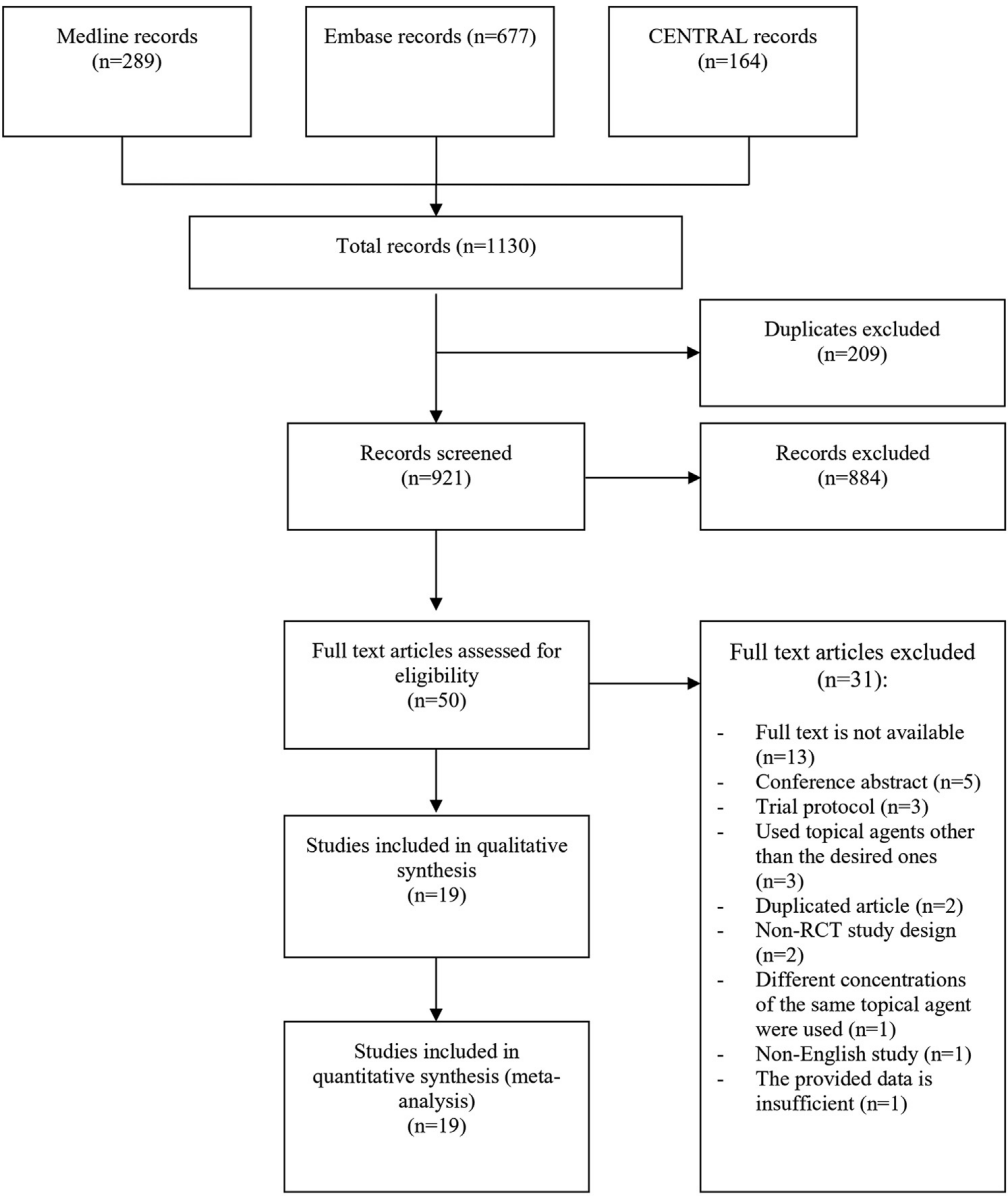
Study flow diagram.

The search resulted in a total of 1130 articles. After removal of duplicates, 921 titles and abstracts remained for screening and 50 articles remained for full text assessment. Of those, 31 were excluded for reasons listed in the PRISMA flow diagram (Fig 1). Eventually, a total of 19 articles met the inclusion and exclusion criteria of this review.^1^^,^^15^, ^16^, ^17^, ^18^, ^19^, ^20^, ^21^, ^22^, ^23^, ^24^, ^25^, ^26^, ^27^, ^28^, ^29^, ^30^, ^31^, ^32^

### Characteristic of studies

A total of 8075 participants were included (Supplementary Table I, available via Mendeley at https://doi.org/10.17632/d6x3ysbytf.3). Majority were females (*n* = 4641; 57.47%), males (*n* = 2007; 24.85%), and (*n* = 1427; 17.68%) not reported. The median age of participants in the included trials was 50.2 (range: 45.9-56.9). Of those, (*n* = 133; 1.65%) received one topical agent on half of the face and another topical agent on the other side of the face (split face). The remaining participants received only one of the desired topical agents applied to the entire face. A total of 1088 (13.47%) participants received topical minocycline 1.5%; (*n* = 124; 1.54%) topical azelaic acid 20%; (*n* = 744; 9.21%) topical metronidazole 0.75%; (*n* = 1622; 20.1%) topical azelaic acid 15%; (*n* = 50; 0.62%) topical metronidazole 1%; (*n* = 92; 1.14%) topical minocycline 1%; (*n* = 171, 2.12%) topical minocycline 3%; (*n* = 1388; 17.18%) topical ivermectin 1%; and (*n* = 2796; 34.62%) received topical vehicle (placebo).

### Risk of bias assessment

Of the 19 included RCT, 7 had an overall low risk of bias, 10 had some concerns, and 2 had an overall high risk of bias. The details of the risk of bias of the included trials are shown in (Supplementary Table II, available via Mendeley at https://doi.org/10.17632/d6x3ysbytf.3).

### Outcome

We performed the network meta-analysis, and it was eligible to calculate the IGA score and the adverse events. However, it was not feasible to perform the network meta-analysis on the remaining outcomes (ie, inflammatory lesion count, RosaQoL score, facial local tolerability, and patient satisfaction) since most of the included RCTs minimally addressed these outcomes.

### Investigators’ Global Assessment (IGA)

A total of 16 trials with 20 treatment arms reported the IGA outcome among patients undergoing treatment for rosacea. Among these trials, Mroweitz, et al and Webster et al were multiarm trials.^19^^,^^20^ The final treatment network consisted of 8 nodes and 20 pairwise comparisons (Supplementary Fig 1, available via Mendeley at https://doi.org/10.17632/d6x3ysbytf.3).

**Fig 2:**
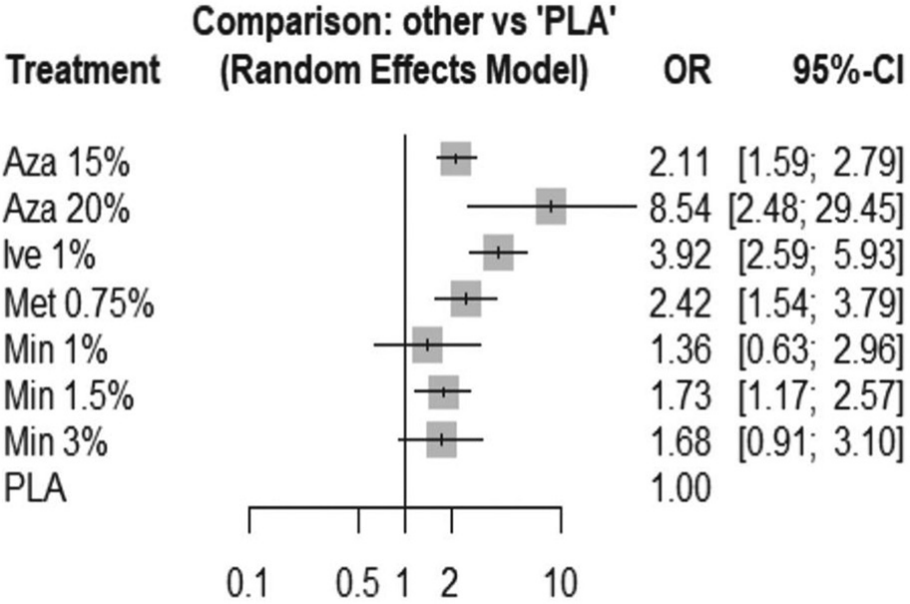
Forest plot comparing topical agents for rosacea with topical vehicle (placebo).

Statistically significant within-design heterogeneity (Q = 18.01, *P* = .01) and between-design inconsistency was noted (Q = 10.06, *P* = .04). Q statistic to assess consistency under the assumption of a full design-by-treatment interaction was nonsignificant (Q = 6.78, *P* = .15). Therefore, the random effects model was used. As per P-scores, azelaic acid 20% yielded the highest effectiveness (*P* = .97), followed by ivermectin 1% (*P* = .88), metronidazole 0.75% (*P* = .62), azelaic acid 15% (*P* = .53), minocycline 1.5% (*P* = .37), minocycline 3% (*P* = .37), minocycline 1% (*P* = .23) and topical vehicle (*P* = .04) (Fig 2). Although azelaic acid 20% yielded the highest odds ratio (OR = 8.54, 95% CI: 2.48-29.45), the 95% confidence interval (CI) was imprecise. Azelaic acid 15%, metronidazole 0.75%, and ivermectin 1% yielded comparable effect sizes, while minocycline 1% and minocycline 3% yielded nonsignificant effect sizes. Only minocycline 1.5% yielded a significant effect size (OR = 1.73, 95% CI: 1.17-2.57).

Back-calculation method to separate indirect from direct evidence did not reveal any disagreements between effect sizes yielded from direct and indirect evidence. Forest plot presenting the pooled effect size from both direct and indirect evidence is presented as (Supplementary Table III, available via Mendeley at https://doi.org/10.17632/d6x3ysbytf.3) and (Supplementary Fig 2, available via Mendeley at https://doi.org/10.17632/d6x3ysbytf.3). Bar graph showing the proportion of direct and indirect evidence is presented as (Supplementary Fig 3, available via Mendeley at https://doi.org/10.17632/d6x3ysbytf.3). Egger’s regression for small study effects was not significant (*P* = .08).

### Risk for adverse effects

The network plot for adverse effect of topical agents for rosacea was reported in 19 studies testing 10 treatments, accounting for 23 pairwise comparisons^1^^,^^15^, ^16^, ^17^, ^18^, ^19^, ^20^, ^21^, ^22^, ^23^, ^24^, ^25^, ^26^, ^27^, ^28^, ^29^, ^30^, ^31^, ^32^ (Supplementary Fig 4, available via Mendeley at https://doi.org/10.17632/d6x3ysbytf.3).

The network meta-analysis was run using random effects due to substantial heterogeneity (I2 = 50.9%; 95% CI: 7.3%-74%) and inconsistency between designs (Q = 13.56. *P* = .02). However, homogeneity was noted for within-design heterogeneity (Q = 10.88, *P* = .14). Furthermore, Q statistic to assess consistency under the assumption of a full design-by-treatment interaction suggested the use of random effects for network meta-analysis (Q = 9.89, *P* = .08).

**Fig 3:**
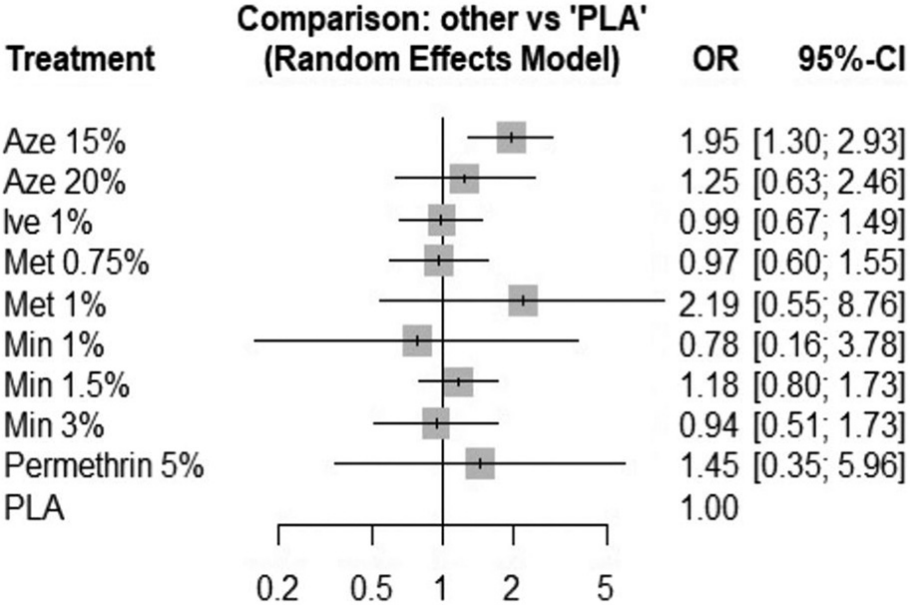
Forest plot comparing adverse events for topical agents with placebo.

As per P-scores, azelaic acid 15% yielded the highest odds for adverse events after treatment (*P* = .14). Compared with topical vehicle (placebo), only azelaic acid 15% yielded statistically significant odds ratio (OR = 1.95, 95% CI: 1.30-2.93), while risk of adverse events for other topical agents was not significantly different (Fig 3). There were significant differences in OR yielded from direct and indirect evidence for 2 comparisons: azelaic acid 15% versus metronidazole 0.75% and azelaic acid 15% versus placebo (Supplementary Table IV and Fig 5, available via Mendeley at https://doi.org/10.17632/d6x3ysbytf.3). Direct estimates comparing azelaic acid 15% and metronidazole 0.75% yielded an OR of 4.56 (95% CI: 1.7-11.94) and from indirect estimates 1.34 (95% CI: 0.67-2.65). The indirect estimate for the comparison between azelaic acid 15% and placebo was substantially higher 5.64 (95% CI: 1.88-16.92) than the direct evidence 1.65 (95% CI: 1.07-2.55). The proportion of studies reporting direct and indirect evidence for each agent is presented as (Supplementary Fig 6, available via Mendeley at https://doi.org/10.17632/d6x3ysbytf.3). Comparison-adjusted funnel plot was symmetric, a finding also corroborated using Egger’s regression (P score = 0.11).

The most commonly reported cutaneous adverse events were face burning or stinging (5.50%), pruritus (2.97%), and application-site pain (2.50%) while the most commonly reported noncutaneous adverse events were upper respiratory tract infection (7.46%) and headache (0.66%) (Supplementary Table V, available via Mendeley at https://doi.org/10.17632/d6x3ysbytf.3).

## Discussion

Since there are multiple treatments available, it is crucial to compare effectiveness, safety, and tolerability. When direct head-to-head data from clinical trials are not available, it is necessary to utilize quantitative analysis to perform indirect comparisons through a network meta-analysis. Our network meta-analysis demonstrated that azelaic acid 20% yielded the highest effectiveness in improving IGA score, followed by ivermectin 1%, metronidazole 0.75%, azelaic acid 15%, minocycline 1.5%, minocycline 3%, minocycline 1%, and placebo respectively.

These results support the conclusion of the most recent Cochrane Collaboration systematic review showing the superior efficacy of azelaic acid compared with metronidazole 0.75% gel for moderate to severe PPR.^33^ Another systematic review showed a high certainty of evidence that topical azelaic acid and topical ivermectin decrease the lesions counts in PPR, and moderate certainty of evidence for topical metronidazole and topical minocycline.^34^ The British “clinical practice guidelines” for rosacea treatment published in 2017 advocated the use of topical treatments metronidazole 0.75% gel or cream or azelaic acid 15% as a first line treatment for PPR if the first-line regimen was unsuccessful, then topical ivermectin cream as a second line. If these topical agents do not work for moderate to severe rosacea, systemic treatment should be considered. First line systemic treatment is oral tetracyclines antibiotic.^35^

Since PPR is a chronic cutaneous disorder, the goal of treatment should consider both the short term and long-term maintenance. A research study that looked at the recurrence of PPR after treatment discontinuation for 6 months found that azelaic acid cream had a significantly lower recurrence score of inflammatory lesions than metronidazole 0.75% cream and permethrin 5% cream, although there was no significant difference in erythema score between the 2 topical agents.^17^ Likewise, a multicenter clinical trial concluded that the majority of patients who used azelaic acid 15% gel alone experienced a lower recurrence rate.^36^ Azelaic acid's effectiveness in the management of rosacea is probably attributable to its multiple mechanisms of actions, which include anti-inflammatory, antimicrobial, antimycotic, and antikeratinizing properties.^37^

In our evaluation of the safety profile in this network meta-analysis, azelaic acid 15% was found to have higher side effects than azelaic acid 20%. This is likely due to the delivery vehicle, as azelaic acid 15% is available in gel or foam formulation while azelaic acid 20% only exists as a cream. The cutaneous penetration and permeation of azelaic acid 15% gel is markedly greater than that of azelaic acid 20% cream.^38^ Another potential explanation is the water-based nature of the gel, which contains no oils or surfactants but instead contains substances that could make it more of an irritant to rosacea patients, who typically have sensitive skin.^15^^,^^29^

Our review showed that the risk for adverse events for other topical agents was not significant, but the majority of the included RCTs in our review concluded that azelaic acid in general exhibits more adverse events compared to the other topical agents or placebo. However, these side effects were tolerable. They included face burning or stinging, pruritus, scaling, dryness, and erythema, with no difference in the side-effects profiles between the 20% cream and the 15% foam formulations.

Furthermore, in this study, minocycline 1% exhibited the least adverse effect rate. Likewise, studies demonstrated that there were no serious treatment-related adverse events. Most adverse events were mild to moderate for minocycline, and pruritus was the most common cutaneous adverse event followed by erythema, mild inflammatory/post inflammatory hyperpigmentation, and mild dryness.^39^

### Limitations

We believe that the pooling of direct and indirect comparisons through a network meta-analysis provides the strongest evidence for the efficacy and safety of the most commonly used topical agents for PPR, namely minocycline, metronidazole, azelaic acid, and ivermectin. Nevertheless, we acknowledge that our review has some limitations. First, there are many clinically important outcomes that were overlooked by most of the included RCTs, including inflammatory lesion count, RosaQoL score, facial local tolerability, and patient satisfaction. This could be an interesting question for future trials to address. Second, some of the topical agents, namely minocycline gel 1%, minocycline gel 3%, azelaic acid 20%, and metronidazole 1%, are under-reported and their efficacy and safety-related data are limited. Lastly, there are discerpinceses on the sample size of minocycline 1%, minocycline 1.5%, minocycline 3%, as the sample size of minocycline 1.5% much higher than minocycline 1% and 3%. That could be attribute to the higher effectiveness of minocycline 1.5%.

## Conclusion

Based on the P-score of this network meta-analysis, topical azelaic acid 20% is most effective in treating moderate-to-severe PPR, achieving clear-to-almost-clear IGA scores. It is followed by topical ivermectin 1% and metronidazole 0.75%, respectively. With respect to safety profile, azelaic acid 15% has the most adverse effects and minocycline 1% has the least. These findings can help physicians select topical agents for moderate-to-severe PPR and will help structure evidence-based guidelines for the treatment of rosacea.

## Conflicts of interest

None disclosed.
